# Validation of low-volume blood collection tubes for routine hematologic testing

**DOI:** 10.5937/jomb0-51438

**Published:** 2024-11-16

**Authors:** Giuseppe Lippi, Loredana Martini, Barbara Cortivo, Chiara Zecchetto, Anna Ferrari

**Affiliations:** 1 University of Verona, Section of Clinical Biochemistry, Verona, Italy; 2 General Hospital of Verona, Service of Laboratory Medicine, Verona, Italy

**Keywords:** preanalytical variability, blood tubes, blood drawing, validation, small volume, preanalitička varijabilnost, epruvete za krv, vađenje krvi, validacija, mali obim

## Abstract

**Background:**

Low-volume blood tubes offer several advantages in facilitating blood collection, reducing iatrogenic anemia and spurious hemolysis, but their clinical reliability must be validated. We planned this investigation for establishing the reliability of routine hematologic testing in low-volume tubes before their implementation into clinical practice.

**Methods:**

Blood was drawn from 44 ostensibly healthy laboratory professionals into three blood tubes, as follows: 3.0 mL of blood into a 3.0 mL K2EDTA standard reference blood tube, 0.5 mL of blood into a second 3.0 mL K2EDTA standard blood tube, and 0.5 mL of blood into a 0.25-0.5 mL K2EDTA low-volume blood tube. Hematologic testing was performed on Sysmex XN-10 hematology analyzer.

**Results:**

Statistically significant differences were observed in total white blood cell count, neutrophil count, lymphocyte count, red blood cell count, platelet count, hemoglobin, hematocrit, mean corpuscular volume (MCV), mean corpuscular hemoglobin concentration (MCHC), and mean platelet volume in both 0.5 mL-filled 3.0 mL standard blood tubes and 0.5 mL-filled low-volume blood tubes. Although none of these variations was found to be clinically significant in the 0.5 mL-filled low-volume blood tube compared to the desirable specifications, hematocrit, MCV and MCHC displayed a clinically significant bias in the 0.5 mL filled 3.0 mL K2EDTA standard blood tube.

**Conclusions:**

These results suggests that K2EDTA low-volume blood tubes could safely replace standard blood tubes for preventing the receipt of insufficient samples, but also for facilitating blood collection in patients with difficult veins and reducing the risk of iatrogenic anemia and spurious hemolysis.

## Abstract (sr)

**Uvod:**

Epruvete za krv male zapremine nude nekoliko prednosti u olakšavanju sakupljanja krvi, smanjenju jatrogene anemije i lažne hemolize, ali njihova klinička pouzdanost mora biti potvrđena. Planirali smo ovo istraživanje radi utvrđivanja pouzdanosti rutinskih hematoloških ispitivanja u epruvetama male zapremine pre njihovog uvođenja u kliničku praksu.

**Metode:**

Krv je uzeta od 44 navodno zdrava laboratorijska stručnjaka u tri epruvete, i to: 3,0 mL krvi u standardnu epruvetu za krv od 3,0 mL K2EDTA, 0,5 mL krvi u drugu od 3,0 mL K2EDTA standardnu epruvetu i 0,5 mL krvi. Krv u epruvetu za krv od 0,25-0,5 mL K2EDTA male zapremine. Hematološko ispitivanje je obavljeno na Sismek KSN-10 hematološkom analizatoru.

**Rezultati:**

Uočene su statistički značajne razlike u ukupnom broju belih krvnih zrnaca, broju neutrofila, limfocita, crvenih krvnih zrnaca, broju trombocita, hemoglobinu, hematokritu, srednjem korpuskularnom volumenu (MCV), srednjoj koncentraciji korpuskularnog hemoglobina (MCHC) i srednjoj koncentraciji trombocita zapremine u obe standardne epruvete od 3,0 mL napunjene 0,5 mL kao i u epruvetama male zapremine napunjenim 0,5 mL. Iako nije utvrđeno da nijedna od ovih varijacija nije klinički značajna u epruveti male zapremine napunjenoj 0,5 mL u poređenju sa poželjnim specifikacijama, hematokrit, MCV i MCHC su pokazali klinički značajnu pristrasnost u standardnoj epruveti za krv od 0,5 mL ispunjenoj od 3,0 mL K2EDTA.

**Zaključak:**

Ovi rezultati sugerišu da bi K2EDTA epruvete male zapremine mogle bezbedno da zamene standardne epruvete za krv za sprečavanje prijema nedovoljnih uzoraka, ali i za olakšavanje prikupljanja krvi kod pacijenata sa teškim venama i smanjenje rizika od jatrogene anemije i lažne hemolize.

## Introduction

Preanalytical variability, usually referred to as the sum of all variations that may occur from blood collection to the actual analysis of the specimens, may have a potential impact on both the accuracy and reliability of test results [1] [2]. Hematologic testing is no exception to this rule, as incorrect or mishandled procedures occurred before sample testing may lead to unreliable data that could ultimately compromise the quality of the clinical decision making [3].

In a retrospective study published almost 15 years ago at an Italian University Hospital, the prevalence and type of preanalytical errors in inpatient samples referred for a complete blood count were analyzed over a one-year period [4]. Of 138,769 tubes received at the local laboratory for routine and stat hematologic analysis, 500 (0.4%) had at least one preanalytical problem, the most common of which was improper clotting (384/500; 76.8%). Nevertheless, the laboratory also detected 35 samples with insufficient volume, accounting for 7% of all preanalytical errors, although the relative frequency increase to over 14% of all preanalytical problems in samples received from pediatric departments. In another recent retrospective study conducted in a tertiary care center in Saudi Arabia, the authors systematically monitored the total number of preanalytical errors in a hematology laboratory over a one-year period [5]. Of the total 67,892 blood samples received, 886 (1.3%) were rejected due to preanalytical problems, and the most frequent preanalytical error was the receipt of insufficient sample volume (480/886; 54.2%), with an almost identical prevalence between inpatient and outpatient samples (i.e., 53.7% vs. 54.7%).

These previous studies provide important evidence that underfilling of blood tubes for hematology testing may be relatively common, especially when samples are drawn from patients with difficult veins or from those in extreme age groups (i.e., neonates, very young children, or elderly patients), in whom iatrogenic anemia due to repeated blood draws may be a relevant issue [6].

Irrespective of samples whose blood volume is so insufficient that the analysis cannot technically be performed, the receipt of insufficiently filled samples can also be a source of problems in hematologic testing. It has recently been shown that spray-dried evacuated K_2_EDTA blood tubes filled to less than 67% of their nominal volume can lead to generation of spurious hematologic test results, especially for some parameters such as the red blood cell count (RBC), hemoglobin (Hb), hematocrit (Ht), mean corpuscular volume (MCV), mean corpuscular hemoglobin concentration (MCHC) and mean platelet volume (MPV) [7]. Since the collection of insufficient samples is relatively common and can lead to unreliable test results of the complete blood cell count, we planned this pilot study to validate the use of K_2_EDTA low-volume blood tubes, designed to collect a significantly smaller volume of blood, compared to »standard« K_2_EDTA blood collection tubes.

## Materials and methods

We recruited 44 ostensibly healthy volunteers (mean age 42±13 years, 35 years) from the personnel of the laboratory medical service at the General Hospital of Verona (Italy). Blood was drawn from peripheral veins of the upper arm by the same skilled phlebotomist (L.M.). The vein was punctured with a 21-gauge, 0.8×20×180 mm disposable butterfly needle (Pic Vacumirage SmartSafe Kit, Pikdare S.p.A., Come, Italy) connected to a 5-mL syringe (Italiana Medicazione S.r.l., Pescara, Italy). To standardize sample preparation, after removing the cap, the blood was gently transferred into three evacuated blood tubes, as follows: 3.0 mL of blood into a 3.0 mL, 13×75 mm, spray-dried K_2_EDTA standard blood tube (Vacutest, Kima, Arzergrande, Padova, Italy; Lot: Z2963), 0.5 mL of blood into a second 3.0 mL, 13×75 mm, spray-dried K_2_EDTA standard blood tube (Vacutest, Kima, Arzergrande, Padova, Italy; Lot: Z2963), and 0.5 mL of blood into a 0.25–0.5 mL, 13×75 mm, spray-dried K_2_EDTA low-volume blood tube (MiniCollect® Complete, Greiner Bio-One, Kremsmünster, Austria; Lot: A230743P). The filling of all three blood tubes was completed 15–30 seconds after the end of venipuncture, all tubes were recapped and mixed carefully by 4 to 6 times inversion. Hematologic testing was then performed 30–45 min after collection using an identical Sysmex XN-10 hematology analyzer (Sysmex Corporation, Kobe, Japan), the characteristics of which have been described in detail elsewhere [8].

The hematologic data used for this study included the total count of white blood cells (WBC) with differential (WBC populations reported in absolute values), RBC, platelet count (PLT), Hb, Ht, MCV, mean hemoglobin content (MCH), MCHC, and MPV. Test results were expressed as median and interquartile range (IQR). Statistical analysis (Analyse-it for Microsoft Excel; Analyse-it Software Ltd, Leeds, UK) was based on Wilcoxon matched-pairs test, while the identification of a clinically significant bias was performed by comparing the percent analyte variations in the two 0.5 mL-filled tubes from the reference 3.0 mL-filled standard tube (using Bland & Altman plots) with the desirable specifications calculated from intra- and inter-individual biological variation, as summarized in Table 1 [9].

**Table 1:** Differences of routine hematologic testing in 3.0 mL K_2_EDTA standard blood tubes filled with 3.0 mL or 0.5 mL of blood and in 0.25–0.5 mL low-volume blood tubes filled with 0.5 mL of blood. Differences from the reference 3.0 mL appropriately filled K_2_EDTA blood tube were assessed with Wilcoxon matched-pairs test, while the percentage difference from the reference 3.0 mL appropriately filled K_2_EDTA blood tube were compared with the analytical quality specifications for desirable bias. Data are shown as median and interquartile range (IQR, i.e., 25-75^th^ percentile). † *P*<0.05; ‡ *P*<0.01. Values exceeding the desirable bias are provided **in bold**.<br>WBC, White Blood Cells count; RBC, Red Blood Cells count; MCV, Mean Corpuscular Volume; MHC, Mean Hemoglobin Content; MCHC, Mean Corpuscular Hemoglobin Concentration; MPV, mean platelet volume.

		**3.0 mL <br>standard tube <br>(3.0 mL filling)**	**3.0 mL standard tube <br>(0.5 mL filling)**		**0.5 mL low-volume tube <br>(0.5 mL filling)**	
		**Median (IQR)**	**Median (IQR)**	**Bias (95%CI)**	**Median (IQR)**	**Bias (95%CI)**
**WBC (×10^9^/L)**	± 5.6%	6.61 (5.55–8.26)	6.48 (5.68–8.32)†	-1.2 (-2.1 to -0.4)%	6.45 (5.45–8.15)‡	-2.2 (-3.1 to -1.3)%
**Neutrophils **<br>**(×10^9^/L)**	± 9.2%	3.65 (3.10–4.54)	3.54 (3.10–4.48)†	-0.9 (-2.0 to 0.2)%	3.56 (3.07–4.47)†	-2.0 (-3.1 to -0.9)%
**Lymphocytes** <br>**(×10^9^/L)**	± 9.2%	2.13 (1.86–2.62)	2.11 (1.83–2.43)†	-1.5 (-2.8 to -0.1)%	2.10 (1.80–2.48)‡	-2.4 (-3.8 to -1.0)%
**Monocytes** <br>**(×10^9^/L)**	± 13.2%	0.50 (0.42–0.62)	0.49 (0.43–0.60)	-1.8 (-3.7 to 0.1)%	0.50 (0.42–0.61)	-0.9 (-3.3 to 1.6)%
**Eosinophils** <br>**(×10^9^/L)**	± 19.8%	0.11 (0.05–0.17)	0.10 (0.06–0.17)	1.0 (-4.6 to 6.6)%	0.09 (0.07–0.17)	4.3 (-8.1 to 16.7)%
**Basophils **<br>**(×10^9^/L)**	± 15.4%	0.04 (0.03–0.06)	0.04 (0.03–0.05)	-1.5 (-10.3 to 7.4)%	0.04 (0.03–0.06)	-6.7 (-16.3 to 2.9)%
**Red Blood Cells** <br>**(×10^12^/L)**	± 1.7%	4.62 (4.29–4.94)	4.67 (4.33–5.00)‡	1.5 (1.1 to 1.8)%	4.63 (4.32–4.97)‡	1.1 (0.5 to 1.7)%
**Platelet count **<br>**(×10^9^/L)**	± 5.9%	262 (215–305)	252 (208–298)‡	-2.4 (-3.4 to -1.4)%	252 (207–286)‡	-4.2 (-6.1 to -2.3)%
**Hemoglobin** <br>**(g/L)**	± 1.8%	135 (129–140)	135 (132–143)‡	1.2 (0.9 to 1.6)%	136 (131–143)‡	1.2 (0.6 to 1.8)%
**Hematocrit**	± 1.7%	0.42 (0.39–0.43)	0.43 (0.41–0.45)‡	**3.8 (3.5 to 4.2)%**	0.42 (0.40–0.44)†	0.8 (0.2 to 1.4)%
**MCV (fl)**	± 1.2%	91.8 (88.5–95.2)	94.5 (90.5–97.4)‡	**2.4 (2.1 to 2.6)%**	91.5 (88.6–94.9)†	-0.2 (-0.4 to -0.1)%
**MCH (pg)**	± 1.4%	30.2 (29.2–31.4)	30.1 (29.0–31.2)	-0.2 (-0.5 to 0.1)%	30.1 (29.4–31.4)	0.2 (-0.2 to 0.5%)
**MCHC (g/L)**	± 0.4%	326 (323–333)	319 (315–323)‡	**-2.6 (-3.0 to -2.2%)**	329 (324–333)†	0.3 (0.0 to 0.6)%
**MPV (fl) **	± 2.3%	10.9 (10.1–11.3)	10.9 (10.3–11.4)†	0.9 (0.1 to 1.6)%	10.9 (10.3–11.6)‡	1.3 (0.6 to 2.0)%

The investigation was performed in accordance with hospital management to validate the reliability of routine hematology tests in low-volume tubes prior to their introduction into clinical practice. Informed consent was obtained from all subjects. The study was conducted in accordance with the Declaration of Helsinki and in compliance with all relevant local legislation and was approved by the Ethics Committee of the University Hospital of Verona (970CESC; July 20, 2016).

## Results

The main results of this study are summarized in Table 1. Compared to the appropriately filled 3.0 mL K_2_EDTA standard blood tube, statistically significant differences were observed for WBC, neutrophils, lymphocytes, RBC, PLT, Hb, Ht, MCV, MCHC and MPV in both the 0.5 mL-filled 3.0 mL K_2_EDTA standard blood tube and in the 0.5 mL-filled low-volume blood tube. However, although none of these variations was found to be clinically significant in the 0.5 mL-filled low-volume blood tube when compared to the desirable specifications, Ht, MCV and MCHC displayed clinically significant variations in the 0.5 mL-filled 3.0 mL K_2_EDTA standard blood tube.

## Discussion

The use of low-volume tubes instead of standard size tubes may have many important beneficial effects in hematologic testing, as demonstrated by some previous studies. For example, Wu et al. used small-volume tubes for blood collection from hospitalized patients [10], and found that this practice was effective to reduce blood loss by more than 25%, that the number of transfusions was also reduced, while an increase in the mean hemoglobin values was also observed in patients admitted to the transplant and critical care units. Similar results were obtained in a subsequent study [11], in which Myles et al. reported that the introduction of small-volume tubes in a tertiary referral hospital was associated with a 42% reduction in blood volume loss, resulting in an overall saving of approximately 180 mL of blood loss over a three-week period. These results were then replicated in another study of patients undergoing major cardiovascular surgery [12], where the authors found that the use of small-volume blood tubes could reduce blood loss to over 800 mL in patients who had been in intensive care for more than 11 days. The recent STRATUS randomized clinical trial, which investigated the effects of small-volume blood tubes on transfusions in the intensive care unit (ICU), also provided interesting findings [13]. The number of RBC units transfused per patient per ICU stay was significantly reduced by 12% (relative risk, 0.88; 95% confidence interval (95%CI), 0.77–1.00) after replacing standard volume tubes with small volume tubes, with an absolute reduction of approximately 10 RBC units/100 patients per ICU stay (95%CI, 0.24–20.76).

Due to the low-pressure gradient between the vein and the evacuated blood tube, the use of low-volume blood tubes may also help to reduce the risk of blood cell injury and thus spurious hemolysis during blood drawing, as shown in the studies by Heiligers-Duckers et al. [14] and Giavarina [15]. In addition to the positive impact on reducing the risk of iatrogenic anemia and in vitro hemolysis, low-volume tubes also have some practical advantages, namely the fact that the additive inside the tube is calibrated to the exact fill volume, thus overcoming the potential bias in test results due to an excess of additive (e.g., K_2_EDTA) compared to the volume of blood inside the tube.

Notwithstanding all these important theoretical results, the introduction of new devices for blood collection, thus including new blood tubes, always requires a specific validation procedure aimed at demonstrating that test results are almost interchangeable between different tubes, especially when these different tubes are used simultaneously in the same facility [16].

The results of our study provide a real-world analysis of the reliability of routine hematology tests in low-volume tubes before they are introduced into clinical practice, as to our knowledge very few comparable findings have been published. This is all the more surprising as a number of other studies were planned to validate the clinical use of low-volume blood tubes for hemostasis testing [17] [18] [19]. The only previous study we could identify on hematologic testing was published by Bozdemir et al. [20] in 2023. The authors compared the results of routine hematologic parameters measured in 40 adult in-patients using three low-volume blood tubes (Microtainer K_2_EDTA, 0.5 mL, 13×75 mm: Becton Dickinson, Franklin Lakes, NJ; MiniCollect K_2_EDTA, 0.25–0.5 mL, 13×75 mm: Greiner Bio-One, Kremsmünster, Austria; Microvette K_3_EDTA, 0.5 mL, 10.8×47.6 mm: Sarstedt AG & Co. KG, Nümbrecht, Germany) with those obtained in 2.0 mL, 13×75 mm K_2_EDTA or 2.6 mL, 13×65 mm K_3_EDTA standard blood tubes (Vacutainer; Becton Dickinson, Franklin Lakes, NJ or S-Monovette; Sarstedt AG & Co. KG, Nümbrecht, Germany, respectively). The authors found satisfactory correlations between the various hematologic parameters measured on a Beckman Coulter DxH 800 (Beckman Coulter Inc., Brea, CA, USA) with the different types of tubes, although some notable differences emerged. In particular, significant differences compared to the reference standard blood tubes were found for RBC, Hb, Ht, PLT, MCV and MCH in Microtainer, for MCV and MCH in MiniCollect, and for WBC, RBC, PLT, MCV and neutrophils in Microvette. Although the mean bias did not generally exceed 2%, variations up to 20% were observed for neutrophils, monocytes, lymphocytes and platelets, up to 8% for RBC, Hb, Ht and WBC, and up to 3% for MCV, MCH, and MCHC (no specific description was given in which type of low-volume blood tubes these higher biases were recorded).

The results of our study show that although some analytically significant variations were observed between the reference, appropriately filled 3.0 mL K_2_EDTA standard blood tubes and the low-volume blood tubes used in our study, none of these variations could be considered clinically significant compared to the desirable specifications calculated from intra- and inter-individual biological variation. In contrast, Ht, MCV, and MCHC showed clinically significant variations in inadequately filled (i.e., 0.5 mL) 3.0 mL K_2_EDTA standard blood tubes, which is consistent with our previous findings [7].

Thus, this validation study suggests that low-volume 0.25–0.5-mL blood tubes could be safely used in our institution to prevent the receipt of insufficiently filled K_2_EDTA standard blood tubes, but also for facilitating blood collection practices in patients with difficult veins and lowering the risk of iatrogenic anemia and spurious hemolysis.

Notably, the size of the low-volume blood tubes used in our study is identical to that of the standard hematological blood tubes (i.e., 13×75 mm), making them suitable for automated cell counting.

## Dodatak

### Conflict of interest statement

All the authors declare that they have no conflict of interest in this work.
